# Endogenous Purification of NR4A2 (Nurr1) Identified Poly(ADP-Ribose) Polymerase 1 as a Prime Coregulator in Human Adrenocortical H295R Cells

**DOI:** 10.3390/ijms19051406

**Published:** 2018-05-08

**Authors:** Erika Noro, Atsushi Yokoyama, Makoto Kobayashi, Hiroki Shimada, Susumu Suzuki, Mari Hosokawa, Tomohiro Takehara, Rehana Parvin, Hiroki Shima, Kazuhiko Igarashi, Akira Sugawara

**Affiliations:** 1 Department of Molecular Endocrinology, Tohoku University Graduate School of Medicine, 2-1 Seiryo-machi, Aoba-ku, Sendai 980-8575, Japan; erika.noro.r7@dc.tohoku.ac.jp (E.N.); mk59040@gmail.com (M.K.); h.shimada.is1@med.tohoku.ac.jp (H.S.); s.suzuki0616@med.tohoku.ac.jp (S.S.); m.hosokawa@med.tohoku.ac.jp (M.H.); ten10104shen@yahoo.co.jp (T.T.); rehanadu@yahoo.com (R.P.); 2 Department of Biochemistry, Tohoku University Graduate School of Medicine, 2-1 Seiryo-machi, Aoba-ku, Sendai 980-8575, Japan; shima4@med.tohoku.ac.jp (H.S.); igarashi@med.tohoku.ac.jp (K.I.)

**Keywords:** aldosterone, Nurr1, PARP1, NR4A2, hypertension, treatment-resistant hypertension, AG14361, HSD3B1, CYP11B2, H295R

## Abstract

Aldosterone is synthesized in zona glomerulosa of adrenal cortex in response to angiotensin II. This stimulation transcriptionally induces expression of a series of steroidogenic genes such as *HSD3B* and *CYP11B2* via NR4A (nuclear receptor subfamily 4 group A) nuclear receptors and ATF (activating transcription factor) family transcription factors. Nurr1 belongs to the NR4A family and is regarded as an orphan nuclear receptor. The physiological significance of Nurr1 in aldosterone production in adrenal cortex has been well studied. However, coregulators supporting the Nurr1 function still remain elusive. In this study, we performed RIME (rapid immunoprecipitation mass spectrometry of endogenous proteins), a recently developed endogenous coregulator purification method, in human adrenocortical H295R cells and identified PARP1 as one of the top Nurr1-interacting proteins. Nurr1-PARP1 interaction was verified by co-immunoprecipitation. In addition, both siRNA knockdown of PARP1 and treatment of AG14361, a specific PARP1 inhibitor suppressed the angiotensin II-mediated target gene induction in H295R cells. Furthermore, PARP1 inhibitor also suppressed the aldosterone secretion in response to the angiotensin II. Together, these results suggest PARP1 is a prime coregulator for Nurr1.

## 1. Introduction

Aldosterone is a steroid hormone which plays a crucial role in maintaining salt/water balance, and consequently blood pressure homeostasis [1]. Aldosterone is synthesized from cholesterol in the zona glomerulosa (ZG) of adrenal gland mainly in response to angiotensin II and serum potassium through a series of chemical reaction steps [2]. Synthesized and secreted aldosterone binds to mineralocorticoid receptor (MR) expressed in aldosterone-targeted tissues such as renal tubule [3], subsequently followed by transcription of targeted genes such as *ENaC*, which regulate the sodium–potassium balance in renal tubule.

Aldosterone biosynthesis is a multistep chemical reaction regulated by a series of enzymes including SCC (cholesterol side-chain cleavage enzyme), 3βHSD (3β hydroxylstroid dehydrogenase), 21-OHase, and AS (aldosterone synthase) [4]. Initially, intracellularly incorporated cholesterols are translocated to the mitochondria by steroidogenic acute regulatory protein (StAR), and then converted to pregnenolone by mitochondorial enzyme SCC (encoded by *CYP11A1*). Cytosolic 3βHSD (encoded by *HSD3B*) converts pregnenolone to progesterone, and followed by conversion to deoxycorticosterone (DOC) by 21-OHase (encoded by *CYP21*). DOC is converted to corticosterone and finally converted to aldosterone by cytosolic AS (encoded by *CYP11B2*). Among these steps, the reaction regulated by StAR and CYP11B2 are believed to be rate-limiting step of aldosterone biosynthesis [5,6]. Recently, Doi et al. proposed that *HSD3B*, especially *HSD3B1* isoform is another rate-limiting enzyme of aldosterone biosynthesis [7], though a controversial result was also reported from another group [8].

Considering the physiological roles of aldosterone, the excess production of the hormone causes a number of pathological outcomes such as primary aldosteronism (PA) [2,9]. PA is characterized by autonomous aldosterone production, and accounts for more than 10% of the patients with hypertension [10]. For treatment of PA, surgical adrenalectomy is applied for patients with unilaterally increased aldosterone production [11], and patients with bilateral increased aldosterone production are treated with drugs such as MR antagonists (spironolactone or eplerenone) [12]. However, a small, but considerable subset of patients remain hypertensive despite administration of these drugs known as treatment-resistant hypertension (TRH), underscoring the need for development of a novel drug [13,14].

The expression levels of the genes of the aldosterone producing enzymes such as *HSD3B1* and *CYP11B2* are transcriptionally regulated in response to angiotensin II. In addition, it is well known that NR4A and ATF family transcription factors such as Nurr1 and ATF2 are responsible for the gene induction [15,16,17]. NR4A family belongs to the nuclear receptor superfamily [18,19,20,21], which are attracting global attention as drug target in variety of diseases [22]. However, the precise molecular mechanism of NR4A-mediated transcription in response to angiotensin II signaling is still largely unclear. Therefore, in present study, we utilized recently developed method called “RIME (rapid immunoprecipitaion mass spectrometry of endogenous proteins)” [23] for identifying Nurr1 (NR4A2)-interacting transcriptional coregulators using human adrenocortical H295R cells as candidates for novel drug target for TRH.

## 2. Results

### 2.1. Purification of Angiotensin II-Induced Nurr1-Associated Proteins in H295R Cells

**Figure 1:**
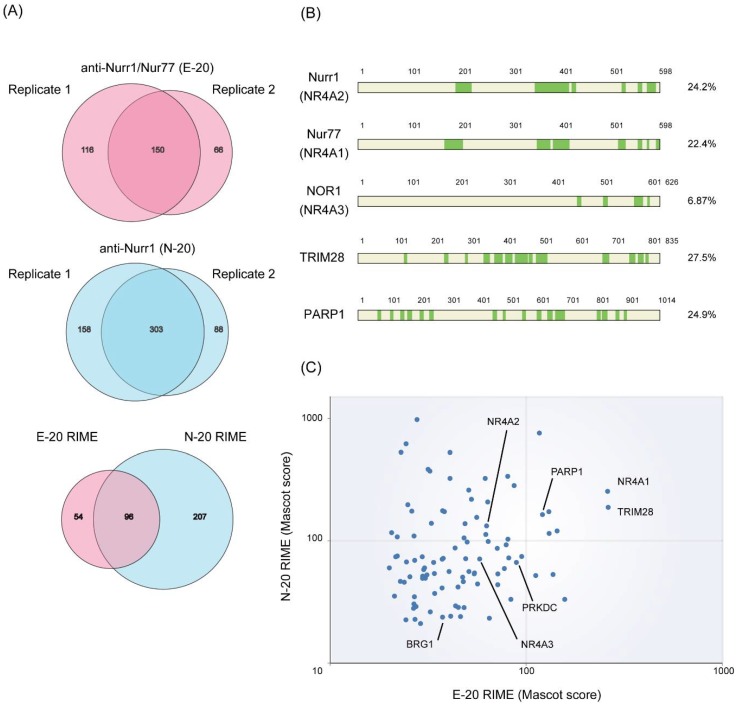
RIME purification of Nurr1-interacting proteins. (**A**) Nurr1 RIME was performed in H295R cells. From two independent RIME purifications using each antibodies indicated, only proteins identified in both experiments were considered and any protein that identified from IgG control was excluded; (**B**) Total peptide coverage of specific identified proteins. Highlighted in green indicates peptides identified with high confidence (False discovery rate (FDR) < 0.01); (**C**) Two replicates of RIME purification using two different antibodies were conducted and proteins identified in all replicates were plotted. The axis represents log_10_ scale of Mascot score for each RIME (replicate 2).

Endogenous Nurr1 proteins were isolated from angiotensin II-stimulated H295R cells using RIME method with two antibodies against Nurr1: E-20 (anti-Nurr1/Nur77) and N-20 (anti-Nurr1). Purified proteins were then subjected to LC-MS/MS (liquid chromatography-tandem mass spectrometry) analysis for protein identification. We used rabbit IgG as a negative control for the purification. From two independent RIME purifications using each antibody, we only considered proteins identified in both experiments and excluded any protein that identified from IgG control (Figure 1A). Peptide coverages of specific identified proteins are shown in Figure 1B. Mascot score of identified proteins in RIME using two antibodies were plotted as shown in Figure 1C (Table S1). Adding to NR4A nuclear receptors (Nurr1, Nur77 and NOR-1) that are known to form heterodimer between NR4A members [24], TRIM28 [25] and BRG-1 [26], reported Nurr1 interacting proteins, were identified with the highest score, indicating successful RIME purification and associated protein identification. Among identified proteins, poly(ADP-ribose) polymerase 1 (PARP1) was one of the top hit proteins (Figure 1C). PARP1 is an enzyme that transfers ADP-ribose groups to its target proteins, and thereby plays a pivotal role in the broad range of biological processes such as DNA damage repair and transcription [27]. As PARP1 is increasingly attracting the attention as promising drug target for cancer therapy [28], we focused on the effect of PARP1 on Nurr1-mediated gene regulation. 

### 2.2. Nurr1 Forms Stable Protein Complex with PARP1

**Figure 2:**
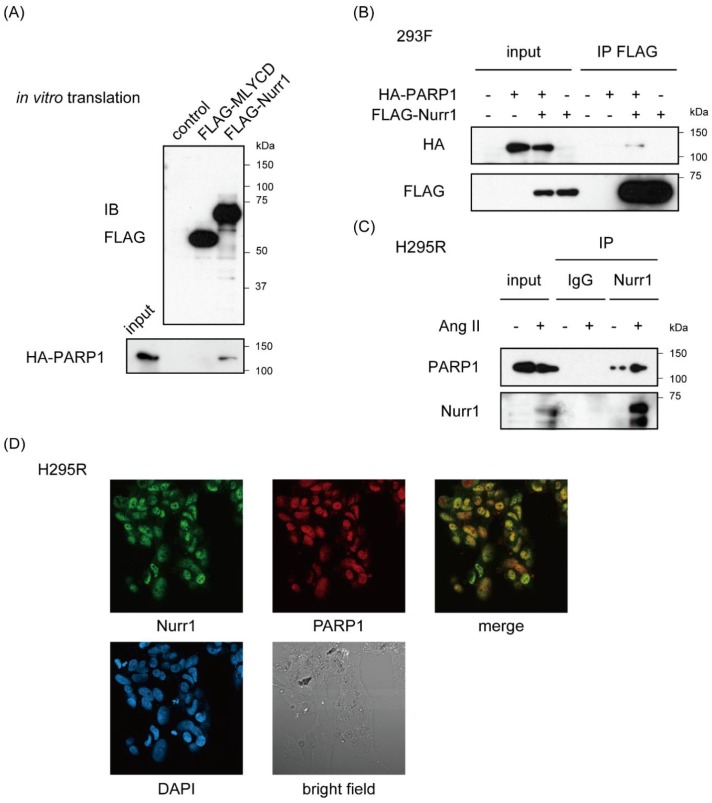
Stable protein complex formation of Nurr1 with PARP1. (**A**) In vitro interaction between FLAG-Nurr1 and HA-PARP1. Confirmation of the expression level of each FLAG-tagged protein was shown on the upper image. Pull-down assay was performed with in vitro-translated proteins. Mixtures of indicated proteins were immunoprecipitated with FLAG-M2 agarose. FLAG-MLYCD protein was used as a negative control. Molecular weights of marker proteins are indicated on the right side; (**B**) Immunoprecipitation of 293F cells exogenously expressing FLAG-Nurr1 and/or HA-PARP1, followed by western blot analysis using indicated antibodies. Molecular weights of marker proteins are indicated on the right side; (**C**) Immunoprecipitation of H295R cells lysates stimulated with 100 nM angiotensin II (AngII) or not for 6 h, followed by western blot analysis using anti-Nurr1 and anti-PARP1 antibodies. Rabbit immunoglobulin (IgG) immunoprecipitates were used as a negative control. Molecular weights of marker proteins are indicated on the right side; (**D**) Confocal immunofluorescence detection of Nurr1 and PARP1 in H295R cells (magnification 40×). H295R cells stimulated with 100 nM angiotensin II (AngII) for 6 h were fixed with formaldehyde and stained with indicated antibodies. DAPI (4′,6-diamidino-2-phenylindole) staining and bright field images of the same area were shown in the lower panael.

NOR-1 (NR4A3) was also known to interact with PARP1 [29]. Given heterodimerization between NR4A members, we could not exclude the possibility that PARP1 was purified with NOR-1 heterodimerized with Nurr1. Therefore, to verify that Nurr1 interacts with PARP1 directly, we conducted pull-down assay using in vitro-translated proteins in cell-free system. In vitro translated FLAG-Nurr1 and hemagglutinin (HA)-PARP1 were mixed and immunoprecipitated with anti-FLAG antibody. FLAG-Nurr1, but not FLAG-tagged malonyl-CoA (coenzyme A) decarboxylase (MLYCD) were co-immunoprecipitated with HA-PARP1, indicating direct interaction between Nurr1 and PARP1 (Figure 2A and Figure S1A). And exogenously expressed FLAG-Nurr1 were also specifically co-immunoprecipitated with HA-PARP1 in 293F cells (Figure 2B and Figure S1B). Furthermore, we conducted immunoprecipitation assay using H295R cell lysates which were stimulated with or without 100 nM angiotensin II for 6 h. As shown in Figure 2C, western blot analysis revealed robust induction of Nurr1 protein in response to angiotensin II. On the other hand, the expression level of PARP1 was not affected by angiotensin II stimulation. Furthermore, angiotensin II-induced endogenous Nurr1 was co-immunoprecipitated with anti-PARP1 antibodies in H295R cells (see also Figure S1C), suggesting that Nurr1 forms a stable protein complex with PARP1 on angiotensin II stimulation consisting with the present result from RIME purification. Immunofluorescence assay for Nurr1 and PARP1 also supports this conclusion (Figure 2D).

### 2.3. PARP1 Supports Nurr1-Mediated Transcription Dependent on Enzymatic Activity

**Figure 3:**
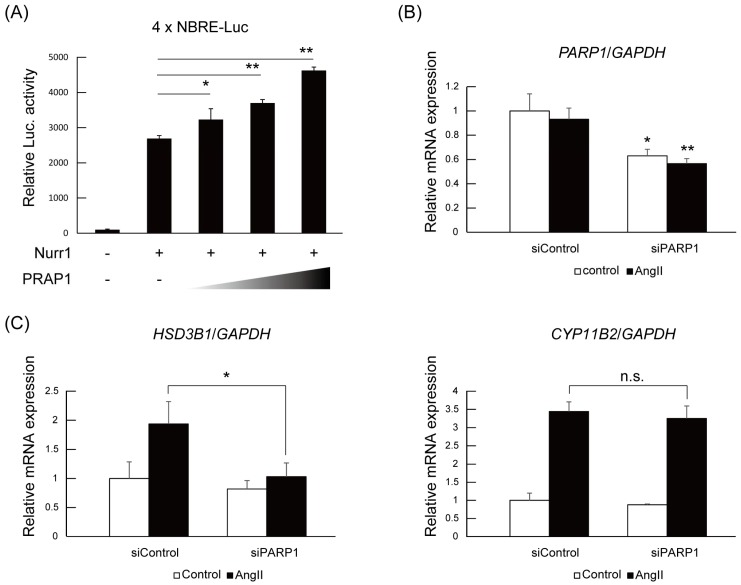

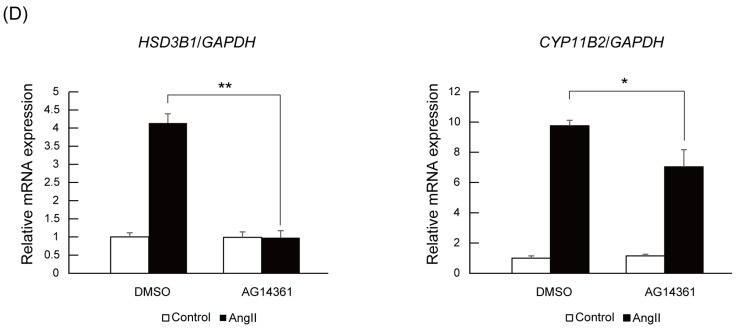
PARP1 is a Nurr1 coactivator in H295R cells. (**A**) Coactivator activity of PARP1 with Nurr1. 293F cells were transiently transfected with pGL4.10 vector (100 ng each), which contains four copies of NBRE sequences, and with FLAG-Nurr1 (50 ng each) and/or HA-PARP1 (0, 50, 100, 250 ng) expression vector. Luciferase (Luc.) activity was normalized to the β-gal internal control. The *p* value was calculated by student’s *t*-test (*n* = 4). The error bars indicate standard deviations. *, *p* < 0.05, **, *p* < 0.01; (**B**) Confirmation of knockdown by siRNA. H295R cells were transfected with siRNAs and incubated for 24 h. After incubation, cells were stimulated with or without 100 nM angiotensin II for 6 h and examined for the expression of PARP1 by quantitative PCR. The expression levels of the *PARP1* gene were normalized to the endogenous *GAPDH* gene. The error bars indicate standard deviations. The *p* value was calculated by Student’s *t*-test (*n* = 3). *, *p* < 0.05, **, *p* < 0.01; (**C**) The effect of PARP1 knockdown on angiotensin II-induced Nurr1 target gene expression was assessed by qPCR. The expression levels of the indicated genes were normalized to the endogenous *GAPDH* gene. The error bars indicate standard deviations. The *p* value was calculated by Student’s *t*-test (*n* = 3). *, *p* < 0.05, n.s., not significant; (**D**) The effect of PARP1 inhibitor AG14361 on angiotensin II-induced Nurr1 target gene expression was assessed by qPCR. Cells were treated with 10 μM of AG14361 and 100 nM angiotensin II for 6 h. The expression levels of the indicated genes were normalized to the endogenous *GAPDH* gene. The error bars indicate standard deviations. The *p* value was calculated by Student’s *t*-test (*n* = 3). *, *p* < 0.05, **, *p* < 0.01.

Next, we investigated the contribution of PARP1 to Nurr1-mediated gene regulation. Firstly, a reporter assay using pGL4.10 reporter plasmid containing four NBRE (NGFI-B response element) sequences in the luciferase gene promoter was performed to determine whether PARP1 acted as a coregulator with Nurr1. As shown in Figure 3A, PARP1 significantly potentiated the transactivation activities of Nurr1 in 293F cells in a dose-dependent manner. Next, we introduced the PARP1 siRNA into the H295R cells and examined the expression levels of Nurr1-targeted genes such as *HSD3B1* and *CYP11B2* [16,30]. PARP1 siRNA and control siRNA were transfected to H295R cells and then stimulated with angiotensin II. The mRNAs were extracted and analyzed by quantitative PCR. As shown Figure 3B, the mRNA levels of *PARP1* were specifically repressed to about 60% of control by the siPARP1. Under the condition of *PARP1* knock down, angiotensin II-induced upregulation of *HSD3B1* mRNA levels was significantly suppressed, although significant suppression of *CYP11B2* induction was not detected (Figure 3C). This target gene preference could be explained by the degree of contribution of Nurr1 to the transcriptional regulation of the genes. While the induction of *HSD3B1* mRNA is only dependent on NR4A NRs, *CYP11B2* gene is regulated by both NR4A NRs and ATF family transcription factors [30]. Therefore siRNA knock down of PARP1 might not be sufficient for repression of *CYP11B2* induction. 

To assess the activity dependency of PARP1 on Nurr1-mediated transcription, we utilized AG14361, a specific inhibitor for PARP1 [31]. As shown in Figure 3D, treatment of H295R cells with 10 μM of AG14361 significantly suppressed angiotensin II-induced HSD3B1 induction. Adding to HSD3B1, induction of CYP11B2 was also significantly suppressed by AG14361 treatment although the degree of suppression is milder than HSD3B1. These data suggest that PARP1 acts as a Nurr1 coactivator dependent on its catalytic activity.

### 2.4. PARP1 Inhibitor Repressed Angiotensin II-Stimulated Aldosterone Secretion in H295R Cells

**Figure 4:**
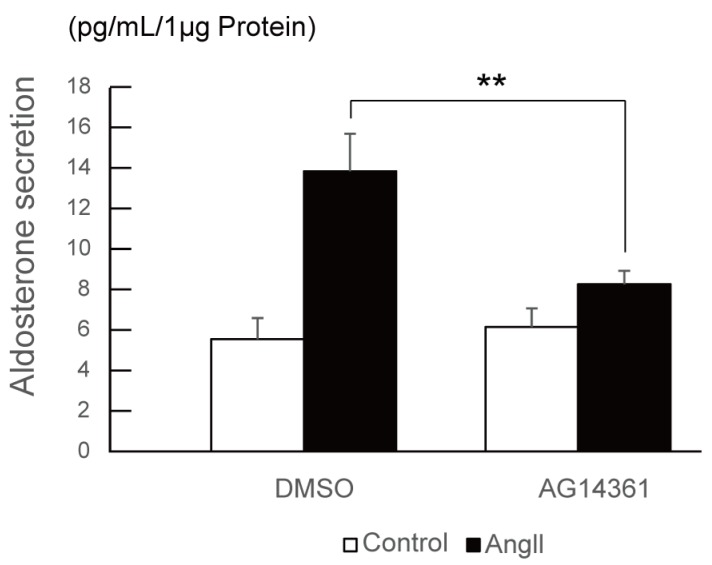
PARP1 inhibitor suppresses aldosterone secretion induced by angiotensin II in H295R cells. The effect of PARP1 inhibitor AG14361 on angiotensin II-induced aldosterone secretion was assessed by qPCR. Cells were treated with 10 μM of AG14361 and 100 nM of angiotensin II for 24 h. Aldosterone was extracted from the conditioned medium of each cells. The concentration of aldosterone measured by ELISA was normalized to the protein content derived from the cells. The error bars indicate standard deviations. The *p* value was calculated by Student’s *t*-test (*n* = 4). **, *p* < 0.01.

Finally, we investigated the effect of PARP1 inhibitor on angiotensin II-stimulated aldosterone secretion in H295R cells. After 24 h of angiotensin II stimulation with or without 10 μM of PARP1 inhibitor, the conditioned medium of H295R cells were collected and hydrophobic fraction was extracted using dichloromethane, and then aldosterone concentration was measured by ELISA (enzyme-linked immunosorbent assay). As shown in Figure 4, PARP1 inhibitor AG14361 significantly suppressed the angiotensin II-stimulated aldosterone secretion in H295R cells, supporting that PARP1 acts as a Nurr1 coactivator.

## 3. Discussion

In the present study, we have identified PARP1 as a Nurr1 coactivator in H295R cells using recently developed protein purification method called RIME. PARP1 belongs to a group of ADP-ribosyl transferase enzymes, which transfer ADP-ribose groups of donor NAD^+^ molecules onto their target proteins, and PARP family proteins control a wide array of cellular processes including DNA repair and transcription [27]. So far, NR (nuclear receptor) coregulation activities of PARP1 have been reported from several groups and the regulation function of PARP1 (coactivation/corepression) is dependent on its binding NRs. For example, on binding to RAR (retinoic acid receptor), LXR (liver X receptor) α/β [32] and FXR (farnesoid X receptor) [33], PARP1 acts as a corepressor dependent or independent on its catalytic activity. On the other hand, PARP1 acts as coactivator when the binding partner is ERα [34]. As for NR4A family NRs, PARP1 has been reported to bind with NOR-1 (neuron-derived orphan receptor 1) (NR4A3) and negatively regulate NOR-1-mediated transcriptional activity via NurRE (nur response element) DNA element independent of its catalytic activity [29]. In this study, we have showed that PARP1 interacted with Nurr1, and from the experiments with siRNA and specific inhibitor, PARP1 acts as coactivator for Nurr1 dependent on the catalytic activity. The determinant of the regulation activity of PARP1 is still largely unclear, and further analysis is required to elucidate it. 

In the Figure 3, the experiment with siRNA-mediated PARP1 knockdown and the inhibition of the enzymatic activity of PARP1 with specific inhibitor showed significant suppression of angiotensin II-induced Nurr1 target gene induction such as *HSD3B1* and *CYP11B2*. However, the extent of the suppression level for the two genes was considerably different. For example, AG14361 completely canceled the angiotensin II-induced *HSD3B1* gene induction, on the other hand, *CYP11B2* induction was significantly but moderately suppressed by the inhibitor. This difference can be explained by the difference of transcriptional regulation mechanism of the each gene. On the angiotensin II stimulation, NR4A family NRs are newly synthesized and activated, and already existing ATF family transcription factors are phosphorylated and activated [30]. Both transcription factors regulate *CYP11B2* gene, however, only NR4A family NRs regulate *HSD3B1* gene which has NBRE consensus sequence in its upstream regulatory region. Consistently, the inhibition of the PARP1 activity or PARP1 knockdown resulted in different level of suppression for *HSD3B1* and *CYP11B2*. Given that PARP1 inhibition significantly suppressed the aldosterone secretion in response to the angiotensin II, this result might support that HSD3B1 is a rate-limiting enzyme for aldosterone biosynthesis.

Until today, a variety of PARP1 inhibitor has been developed and clinically used for ovarian, breast and other carcinomas with promising efficiency, and furthermore, new inhibitors such as veliparib and talazoparib have been entered clinical trials [28]. Concerning blood pressure, a number of studies have shown that high doses of nicotinamide, a primitive PARP1 inhibitor, can decrease blood pressure in mice, rats and dogs [35]. As for specific PARP1 inhibitor AG14361, Calabrese et al. reported that AG14361 showed vasoactivity that relaxed vascular smooth muscle, though detailed blood pressure data are not available so far [36]. However, from the viewpoint of the side effect, PARP1 inhibitors are still less acceptable to patients with hypertension. Therefore, further molecular studies are required to specifically inhibit the PARP1 activity for NR4A coregulation in adrenal cortex. For example, a synthetic peptide or compound which can inhibit the interaction between PARP1 and Nurr1 might be one strategy to try.

The identification of responsible substrate for PARP1-mediated Nurr1 coregulation is also of importance for the understanding of the regulation mechanisms. Several PARP1 substrates in transcriptional regulation have been reported including TLE corepressor comlex, a mediator coregulator complex, a condensin I/XRCC1 (X-ray repair cross-complementing protein 1) repair complex, a macroH2A1.1 nucleosome complex and CTCF (CCCTC-binding factor) insulator complex [37]. The role of these factors should be tested in angiotensin II-induced Nurr1 transcriptional regulation. Furthermore, Proteomic approach for ADP-ribosylated substrates on angiotensin II stimulation using recently developed method [38] is also requisite in the near future to clarify the mechanism of PARP-1-mediated Nurr1 transcriptional regulation.

## 4. Materials and Methods 

### 4.1. Reagents

Angiotensin II was purchased from Sigma-Aldrich (St Louis, MO, USA). AG14361 was purchased from Selleck (Houston, TX, USA) and dissolved in Dimethyl sulfoxide (DMSO). siRNAs were purchased from GE Healthcare (Uppsala, Sweden). Following antibodies were used: anti-Nurr1/Nur77 (Santa Cruz Biotechnology, Inc., Santa Cruz, CA, USA, sc-990) and anti-Nurr1 (Santa Cruz Biotechnology, Inc., sc-991) for RIME, anti-Nurr1 (Perseus Proteomics, Tokyo, Japan, PP-N1404-00), anti-PARP1 (Transduction Laboratories, Lexington, KY, USA, P76420), anti-FLAG tag (Sigma-Aldrich, F1804) and anti-HA tag (ICL, Portland, OR, USA, RHGT-45A-Z) for western blot, anti-Nurr1 (Santa Cruz Biotechnology, Inc., sc-991) for immunoprecipitation, anti-Nurr1 (Atlas Antibodies, Bromma, Sweden, HPA000543) and anti-PARP1 (Transduction Laboratories, P76420) for immunostaining, Alexa Fluor 488 goat anti-rabbit IgG (H + L) (Thermo Fisher Scientific, Pittsburgh, PA, USA, A11034) and Alexa Fluor 594 goat anti-mouse IgG (H + L) (Thermo Fisher Scientific, A11032) for 2nd antibodies for immunostaining. For immunoprecipitation of FLAG-tagged protein, Anti-FLAG M2 affinity agarose gel (Sigma-Aldrich, A2220) were used.

### 4.2. Cell Culture

H295R cells (ATCC No. CRL-2128) were obtained from the ATCC (Baltimore, MD, USA) and cultured in D-MEM/Ham’s F-12 (Wako, Osaka, Japan) supplemented with 10% fetal bovine serum (Thermo Fisher Scientific), Insulin-Transferin-Selenium-G Supplements (Thermo Fisher Scientific), 1.25 mg/mL bovine serum albumin (Sigma-Aldrich), 5.35 μg/mL linoleic acid (Sigma-Aldrich) and antibiotics (100 units/mL Penicillin G and 100 μg/mL Streptomycin, Wako). The cells were grown at 37 °C in 5% CO_2_. For angiotensin II stimulation, the culture medium was exchanged to the freshly prepared low glucose D-MEM (Wako) containing 1% charcoal-treated FBS and antibiotics with/without 100 nM angiogtensin II.

293F cells (Thermo Fisher Scientific, R79007) were maintained in D-MEM (Wako) supplemented with 10% fetal bovine serum and antibiotics. For transfection, we used PEI MAX (Polysciences, Inc., Warrington, PA, USA) according to the manufacturer’s instructions.

### 4.3. RIME

RIME experiment was performed as previously reported [23]. Briefly, 5 × 10^7^ cells were stimulated with Angiotensin II for 4 h and then fixed with 1% formalin for 8 min at 37 °C. 2.5 mM glycine was added to final concentration of 100 mM for reaction stop. After cold PBS (phosphate buffered saline) wash, cells were lysed with 10 mL of LB1 buffer (50 mM Hepes pH 7.6, 140 mM NaCl, 1 mM EDTA (ethylenediaminetetraacetic acid), 10% Glycerol, 0.5% NP-40 and 0.25% Triton X-100). Unsolved pellet was then dissolved in 10 mL of LB2 buffer (10 mM Tris pH 8.0, 200 mM NaCl, 1 mM EDTA and 0.5 mM EGTA (ethylene glycol tetraacetic acid)). Unsolved pellet was then dissolved in 300 μL of LB3 buffer (10 mM Tris pH 8.0, 100 mM NaCl, 1 mM EDTA, 0.5 mM EGTA, 0.1% Na-deoxycholate and 0.5% *N*-lauroylsarcosine) and sonicated by handy sonicator (model UR-20P, TOMY SEIKO, Tokyo, Japan, level 8, 20 s, 8 times). After adding 30 μL of 10% Triton X-100, supernatants were mixed with antibody-dynabeads complex and incubated at 4 °C over night. Antibody-dynabeads complex was then washed with RIPA (radio-immunoprecipitation assay) buffer 10 times and twice with AMBIC (100 mM NH_4_HCO_3_), and mixed with 10 μL of 10 ng/μL of trypsin gold (Promega, Madison, WI, USA) and incubated at 37 °C over night. Next day, further 10 μL of trypsin gold was added and incubated for 4 h. Digested peptides were cleaned up using Pierce Detergent Removal Spin Colmn (Thermo Fisher Scientific) and analyzed by LTQ-Orbitrap Velos (Thermo Fisher Scientific) [39,40]. For protein identification, spectra were processed with Proteome Discoverer version 1.3 (Thermo Fisher Scientific) using Mascot algorithm against the human protein database from SwissProt. Peptide data were filtered using a Mascot significance threshold less than 0.05.

### 4.4. In Vitro Translation and Pull down Assay

For pull-down assays, FLAG-Nurr1, HA-PARP1 and FLAG-MLYCD were translated in vitro using Human Cell-Free Protein Expression System (Takara Bio, Tokyo, Japan). FLAG-tagged proteins were mixed with HA-PARP1 for 1h at room temperature and then immunoprecipitated with Anti-FLAG M2 affinity agarose gel (Sigma-Aldrich, A2220). Purified proteins were subjected to western blotting.

### 4.5. Western Blotting

Protein extracts, separated by SDS-PAGE and transferred onto PVDF membranes (Millipore, Bedford, MA, USA), were probed with antibodies against indicated proteins. Proteins of interest were detected with HRP-conjugated donkey anti-rabbit IgG antibody (GE Healthcare) and developed with the Clarity Western ECL Substrate (Bio Rad, Hercules, CA, USA) and BioMax XAR film (Kodak, Rochester, NY, USA).

### 4.6. Immunostaining 

H295R cells were fixed with 3.5% formaldehyde for 15 min at room temperature. After blocking with 1% skim milk, cells were incubated with indicated primary antibodies for 1 h at room temperature. The cells were subsequently washed and incubated with Alexa-coupled 2nd antibodies. Images of stained cells were taken using an LSM800 confocal microscope (Zeiss, Oberkochen, Germany) Dilution rates of antibodies used in this study were as follows: anti-Nurr1 (Atlas Antibodies) and anti-PARP1 (Transduction Laboratories), 1/100, and Alexa Fluor 488 goat anti-rabbit IgG (H + L) and Alexa Fluor 594 goat anti-mouse IgG (H + L), 1/500.

### 4.7. Luciferase Assay

293F cells were transfected with the indicated plasmids using PEI MAX transfection reagents (Polysciences). The total amount of plasmids was adjusted by supplementing it with empty vector. After 48 h incubation, cells were washed with ice cold PBS, and the extracts were prepared using Glo Lysis Buffer (Promega). Luciferase activity was measured using Bright-Glo reagents (Promega). β-galactosidase activity was used as a reference to normalize transfection efficiencies in all experiments.

### 4.8. RNA Isolation and Quantitative Real Time PCR

Total RNA was extracted by Sepasol-RNA I Super G (Nacalai Tesque, Kyoto, Japan) and cDNA was synthesized using PrimeScript Reverse Transcriptase (Takara Bio). Total RNA (500 ng) was used for reverse polymerase chain reaction (PCR) using a Thermal Cycler Dice Gradient (Takara Bio). Quantitative RT-PCR (qPCR) was performed with the CFX Connect Real-Time system (Bio Rad) according to the manufacturer’s instructions. The primer and TaqMan probe sequences for each gene were listed in Table 1. RNA levels were normalized using *GAPDH* gene as an internal standard.

### 4.9. Measurement of Aldosterone Concentration

Measurement of aldosterone concentration was performed as previously described [14,41,42]. H295R cells were plated in 24-multi well plates. After 48 h, culture medium was exchanged to freshly prepared low glucose D-MEM (Wako) containing 1% charcoal-treated FBS and antibiotics with/without 100 nM angiogtensin II and 10 μM AG14361 for 24 h. The aldosterone concentrations of each media were measured by Aldosterone EIA kit (Cayman Chemical, Ann Arbor, MI, USA) according to the manufacture’s instruction. The obtained data were normalized by the protein concentration measured by Protein Assay Kit (Bio Rad). 

## 5. Conclusions

From the results presented here, PARP1 interacts with Nurr1 directly, and coatcivates transactivation function of Nurr1 dependent on its poly(ADP-ribosyl)ation activity. Furthermore, PARP1 is essential for angiotensin II-induced aldosterone secretion in H295R cells. Therefore, we concluded that PARP1 acts as a prime coregulator for Nurr1 in H295R cells.

## Figures and Tables

**Table 1:** Primer and TaqMan probe sequences for quantitative real time polymerase chain reaction (PCR).

Target Gene	Sequence
*hGAPDH*_F	atcccatcaccatcttccag
*hGAPDH*_R	atgagtccttccacgatacc
*hCYP11B2*_F	ggcagaggcagagatgctg
*hCYP11B2*_R	cttgagttagtgtctccaccagga
*hCYP11B2*_probe	(FAM) ctgcaccacgtgctgaagcact (TAM)
*hHSD3B1*_F	agaagagcctctggaaaacacatg
*hHSD3B1*_R	taaggcacaagtgtacagggtgc
*hHSD3B1*_probe	(FAM) ccatacccacacagc (TAM)
*hPARP1*_F	gctcctgaacaatgcagaca
*hPARP1*_R	cattgtgtgtggttgcatga

## Supplementary Materials

Supplementary materials can be found at http://www.mdpi.com/1422-0067/19/5/1406/s1.

## Abbreviations

PARP1	poly(ADP-ribose) polymerase 1
ZG	zona glomerulosa
MR	mineralocorticoid receptor
StAR	steroidgenic acute regulatory protein
DOC	deoxycorticosterone
PA	primary aldosteronism
TRH	treatment-resistant hypertension
RIME	rapid immunoprecipitation mass spectrometry of endogenous proteins
AngII	angiotensin II
IgG	immunogloblin G
DMSO	dimethyl sulfoxide
AMBIC	ammonium bicarbonate
NBRE	NGFI-B responsive element
PCR	polymerase chain reaction
