# Supplementary material for: Endogenous Purification of NR4A2 (Nurr1) Identified Poly(ADP-Ribose) Polymerase 1 as a Prime Coregulator in Human Adrenocortical H295R Cells
Source: Int J Mol Sci. 2018 May 8;19(5):1406. doi: 10.3390/ijms19051406 (PMC5983848; doi:10.3390/ijms19051406)
Supplement: Supplementary file 1 [file ijms-19-01406-s001.zip › Supplementary materials.pdf]

Supplementary Materials: Endogenous Purification of NR4A2 (Nurr1) Identified Poly(ADP-Ribose) Polymerase 1 as a Prime Coregulator in Human Adrenocortical H295R Cells

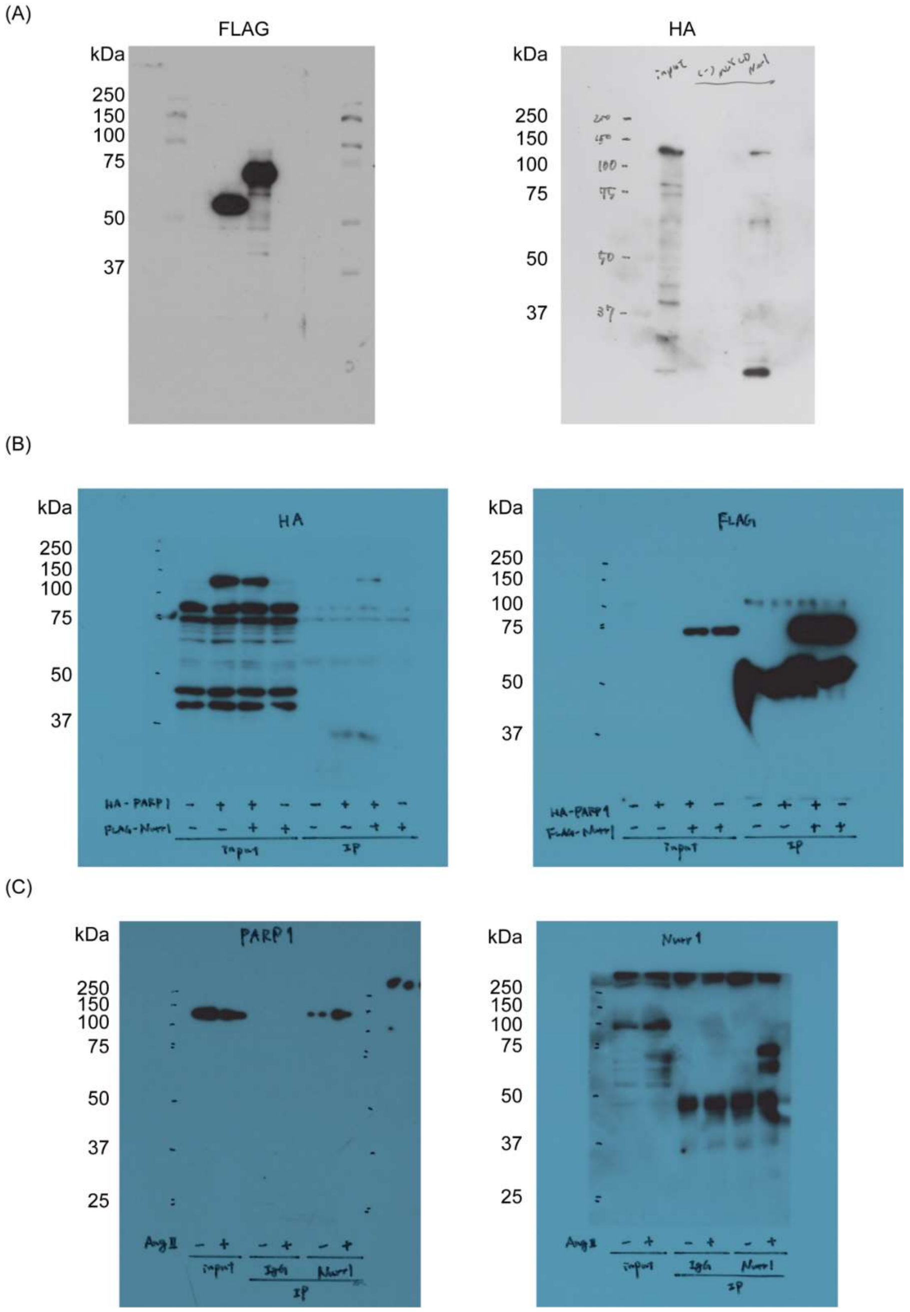

Figure S1. Uncropped scans of western blots used in Figure 2. (**A**) Uncropped scans of Figure 2A. (**B**) Uncropped scans of Figure 2B. (**C**) Uncropped scans of Figure 2C. Molecular weights of marker proteins are indicated on the left side.

Table S1: A full list of identified proteins in Nurr1 RIME
